# Technical Note: Dosimetric evaluation of Monte Carlo algorithm in iPlan for stereotactic ablative body radiotherapy (SABR) for lung cancer patients using RTOG 0813 parameters

**DOI:** 10.1120/jacmp.v16i1.5058

**Published:** 2015-01-08

**Authors:** Damodar Pokhrel, Rajeev Badkul, Hongyu Jiang, Parvesh Kumar, Fen Wang

**Affiliations:** ^1^ Department of Radiation Oncology The University of Kansas Cancer Center Kansas City KS USA

**Keywords:** lung cancer, SBRT, heterogeneity, Monte Carlo, XVMC algorithm, RTOG 0813

## Abstract

For stereotactic ablative body radiotherapy (SABR) in lung cancer patients, Radiation Therapy Oncology Group (RTOG) protocols currently require radiation dose to be calculated using tissue heterogeneity corrections. Dosimetric criteria of RTOG 0813 were established based on the results obtained from non‐Monte Carlo (MC) algorithms, such as superposition/convolutions. Clinically, MC‐based algorithms are now routinely used for lung SABR dose calculations. It is essential to confirm that MC calculations in lung SABR meet RTOG guidelines. This report evaluates iPlan MC plans for SABR in lung cancer patients using dose‐volume histogram normalization per current RTOG 0813 compliance criteria. Eighteen Stage I‐II non‐small cell lung cancer (NSCLC) patients with centrally located tumors, who underwent MC‐based lung SABR with heterogeneity correction using X‐ray Voxel Monte Carlo (XVMC) algorithm (BrainLAB iPlan version 4.1.2), were analyzed. Total dose of 60 Gy in 5 fractions was delivered to planning target volume (PTV) with at least V100%=95%. Internal target volumes (ITVs) were delineated on maximum intensity projection (MIP) images of 4D CT scans. PTV (ITV+5 mm margin) volumes ranged from 10.0 to 99.9 cc (mean=36.8±20.7 cc). Organs at risk (OARs) were delineated on average images of 4D CT scans. Optimal clinical MC SABR plans were generated using a combination of non‐coplanar conformal arcs and beams for the Novalis‐TX consisting of high definition multileaf collimators (MLCs) and 6 MV‐SRS (1000MU/min) mode. All plans were evaluated using the RTOG 0813 high and intermediate dose spillage criteria: conformity index (R100%), ratio of 50% isodose volume to the PTV (R50%), maximum dose 2 cm away from PTV in any direction ($D_{2\text{cm}}$), and percent of normal lung receiving $20\;\text{Gy}\;(V_{20})$ or more. Other organs‐at‐risk (OARs) doses were tabulated, including the volume of normal lung receiving $5\;\text{Gy}\;(V_{5})$, maximum cord dose, dose to <15 cc of heart, and dose to <5 cc of esophagus. Only six out of 18 patients met all RTOG 0813 compliance criteria. Eight of 18 patients had minor deviations in R100%, four in R50%, and nine in $D_{2\text{cm}}$. However, only one patient had minor deviation in $V_{20}$. All other OARs doses, such as maximum cord dose, dose to <15 cc of heart, and dose to <5 cc of esophagus, were satisfactory for RTOG criteria, except for one patient, for whom the dose to <15 cc of heart was higher than RTOG guidelines. The preliminary results for our limited iPlan XVMC dose calculations indicate that the majority (i.e., 2/3) of our patients had minor deviations in the dosimetric guidelines set by RTOG 0813 protocol in one way or another. When using an exclusive highly sophisticated XVMC algorithm, the RTOG 0813 dosimetric compliance criteria such as R100% and $D_{2\text{cm}}$ may need to be revisited. Based on our limited number of patient datasets, in general, about 6% for R100% and 9% for $D_{2\text{cm}}$ corrections could be applied to pass the RTOG 0813 compliance criteria in most of those patients. More patient plans need to be evaluated to make recommendation for R50%. No adjustment is necessary for OAR dose tolerances, including normal lung $V_{20}$. In order to establish new MC specific dose parameters, further investigation with a large cohort of patients including central, as well as peripheral lung tumors, is anticipated and strongly recommended.

PACS number: 8087

## I. INTRODUCTION

SABR with hypofractionated dose schemata has emerged a viable alternative treatment for medically inoperable early‐stage lung cancer patients.^(1)^ Treatment planning for the SABR lung case is challenging due to the involvement of small fields and low‐density lung medium (air), which could result in electronic disequilibrium in the regions near low‐density heterogeneity interface.^(2)^ Dose calculation algorithms in commercial treatment planning systems (TPS) must have accurate modeling for the tissue heterogeneity corrections in order to avoid inaccurate delivery of the monitor units (MUs) of radiation during the patient treatment.

Radiation Therapy Oncology Group (RTOG) 0813 SABR lung protocol requires the participating institutions to generate the treatment plans using dose calculation algorithms that can compute the dose applying tissue heterogeneity corrections. Dosimetric criteria of RTOG 0813 were established based on the results obtained from non‐Monte Carlo (MC) algorithms, such as superposition/convolutions.^3^, ^4^ Recently, several commercial TPS have employed MC‐based dose calculation algorithms, and many researchers have investigated whether MC‐based dose calculation algorithms can meet the dosimetric criteria of RTOG 0813.^5^, ^6^ For instance, Li et al.^(5)^ evaluated the MC algorithm employed in Monaco TPS (Computerized Medical System, St. Louis, MO) for SBRT lung plans and compared the results against the superposition algorithm in XiO TPS (Computerized Medical System, St. Louis, MO). In one of the most recent studies, Rana et al.^(6)^ evaluated the MC‐based Acuros XB employed in Eclipse TPS (Varian Medical Systems, Palo Alto, CA) for the SBRT lung cases, and compared the results against the anisotropic analytical algorithm (AAA) in Eclipse TPS. Li et al.^(5)^ reported that dosimetric results from MC algorithm in Monaco had larger values compared to the ones from superposition algorithm in XiO TPS. In the study by Rana et al.,^(6)^ it was found that Acuros XB resulted in lower magnitudes of R100%, R50%, and $D_{2\text{cm}}$ by 5%, 1.2%, and 1.6%, respectively, on average, than the AAA, except for the normal lung tissue ($V_{20}$) where it was higher by 1.1%. In addition to the variation in dose calculation algorithms and TPS used between these two studies the treatment planning techniques were different as well. Specifically, Li et al.^(5)^ used intensity modulated radiation therapy (IMRT) and Rana et al.^(6)^ used volumetric modulated arc therapy (VMAT), which is referred as RapidArc in Eclipse TPS.

MC is more complex and accurate in handling of tissue heterogeneities. This is because of their ability to more accurately simulate radiation transport of a) secondary scatter photons, and b) lateral electron equilibrium in the MC algorithms, and therefore, more accurately predict dose distribution, specifically, at the low‐density lung and heterogeneous tissues interfaces. That could actually emulate actual measured doses. Clinically, MC‐based algorithms are now routinely used for lung SABR dose calculations. It is essential to confirm that MC calculations in lung SABR meet RTOG guidelines. At our institution, we currently use X‐ray Voxel Monte Carlo (XVMC) algorithm ^(7)^ (BrainLAB iPlan, Feldkirchen, Germany) for dose calculations for our lung SABR patients. To our best knowledge, dosimetric evaluation of iPlan MC lung SABR plan using RTOG 0813 criteria is yet to be reported. One of the requirements of RTOG 0813 protocol is the plan normalization to be done to a single point (e.g., an isocenter or a point inside the planning target volume (PTV)).^3^, ^4^ Since, dose‐volume (DV) normalization is one of the most popular methods used to report the clinical dosimetric results, the main purpose of this paper was to investigates whether those RTOG 0813 dosimetric criteria can be met or not. The results were presented by applying iPlan XVMC algorithm dose calculation and DV normalization method for the clinical SABR lung plans.

## II. MATERIALS AND METHODS

### A. Dose calculation algorithm

Both the pencil beam (PB) and XVMC algorithms were commissioned and clinically implemented in the BrainLAB iPlan RT (version 4.1.2) TPS in our institution. The XVMC was based on the X‐ray Voxel Monte Carlo algorithm ^(7)^ which consists of source modeling, beam collimating system modeling, and patient dose computation. The dose calculation parameters for XVMC in iPlan are spatial resolution, mean variance, dose result type, and MLC model. The spatial resolution defines the size of the dose calculation grid, whereas the mean variance estimates the statistical uncertainty of the MC dose calculation. Choosing smaller variance, the dose calculations would be more accurate at the cost of computation time. Dose to water and to medium are the two options in the dose type; in this calculation it was set to dose to the medium. Readers are advised to refer to the BrainLAB Technical Reference Guide^(8)^ for more details for XVMC algorithm and its clinical implementation.

### B. Verification of XVMC algorithm

There have been several studies focused on verifying Monte Carlo dose calculations in both homogenous and heterogeneous mediums. These studies utilized iPlan RT dose planning system. For instance, using a 6 MV photon beam with a micro‐MLC, an experimental validation of the XVMC algorithm^(9)^ was performed. The validation utilized film, ion chambers, water phantoms, and heterogeneous solid water slabs. These solid water slabs contained bone and lung density equivalent materials. The experiment demonstrated that the calculated dose agreed with the measured dose within ±2% in high‐dose regions, and 2 mm in high‐gradient regions.^9^, ^10^ The average 1D gamma values did not exceed 0.3 with 2%/2 mm creation when comparing calculated and measured dose distributions.^(11)^ Another experimental investigation of XVMC algorithm was presented by Fippel et al.,^(12)^ utilizing a diamond detector and film measurements on homogenous and inhomogeneous phantoms, and 6 MV photons. Fippel and colleagues also demonstrated that the measured and calculated doses agreed within experimentally uncertainty (within ±2%). Dobler et al.,^(13)^ using GAFCHROMIC EBT films, revealed that XVMC predicted the measured dose more accurately with a maximum difference of −3%. However, the PB algorithm overestimated the dose by up to 15% compared to the measurement, suggesting that PB algorithms are limited at the tissue/lung interfaces, and MC is the most accurate algorithm for dose prediction.

Another dosimetric study reported by Chen et al.^(14)^ suggested that XVMC algorithm agreed well with film measurements when using 15 MV photons (<1% difference in lateral profile). Whereas the deviation between the PB algorithm and film measurements was again up to 15%, this is consistent with other studies. In the same study using 35 lung cancer patients, the largest differences were reported for small lung tumors circumferentially encompassed by the lung tissues. The PTV mean dose difference between PB and XVMC was in excess of 4 Gy (prescription dose, 30 Gy). The volume covered by the 9 Gy isodose of lung dose was 8% higher when calculated by XVMC compared with PB in one patient. In addition, Chen and colleagues reported that the dose calculation accuracy is also dependent on tumor size and location. In most recent studies by Sethi et al.,^(15)^ XVMC algorithm was benchmark using ion chamber and EDR films in various depth in five different phantoms with four different density materials. These include tissue‐equivalent plastic water, lung‐equivalent (low‐ and high‐density) corks, and bone, and irradiated with 6 MV photons. For heterogeneous lung phantoms, there was an excellent agreement (<3%) between measured and calculated dose profiles. However, PB calculations significantly overestimated mean PTV measured dose by up to 34%. Measured and PB calculated dose difference increased with decreasing field size, decreasing density, and increasing depth within heterogeneity (however similar results beyond heterogeneity). In contrast, large underestimated dose (up to 50%) was observed in the penumbra region while using PB algorithm.^(15)^ Using a virtual phantom and patients datasets, Miura et al.^(16)^ have also presented similar results in which the mean PTV dose was nearly 20% higher on both the phantom and patients with PB algorithm compared to XVMC.

Our own clinical experience^(17)^ on validating and clinically implementing iPlan XVMC algorithm using Quasar (Modus Medical Devices Inc., London, Canada) phantom study had shown an excellent agreement (within ±2%) between doses calculated using XVMC versus ion chamber measurements for 6 MV‐SRS beam in lung‐equivalent material. In our phantom study,^(17)^ the dose deviation between heterogeneities‐corrected PB and measured value was as large as 9%. MC algorithm not only predicted accurate dose at isocenter, but also at borders of tumors where heterogeneities‐corrected PB overestimated the doses. That is due to the lack of electronic equilibrium in the regions near low‐density tissues heterogeneities interface. In our most recent clinical study^(18)^ with 10 lung cancer patients, the mean PTV dose was as high as 13%, on average, when using heterogeneities‐corrected PB algorithm compared to XVMC using same beam configurations, MLCs, and the same number of MUs. However, the volume covered by 5 Gy, 10 Gy, and 20 Gy isodose lines of the normal lung were comparable (within 3.0%), on average, when calculated by heterogeneities‐corrected PB compared to XVMC. All the peer reviewed literatures cited in this paper have well‐documented the fact that PB calculation overestimates the PTV and OARs doses significantly, as reported above.

### C. Patient simulation and target contouring

Eighteen centrally located Stage I‐II non‐small cell lung cancer (NSCLC) patients who underwent MC‐based lung SABR at the University of Kansas Hospital, Kansas City, KS were included in this retrospective study. The simulation was performed on a 16 slice Phillips Brilliance Big Bore CT Scanner (Philips Healthcare, Andover, MA) and patient in the supine position was immobilized using BlueBAG BodyFIX system (Medical Intelligence, Schwabmuenchen, Germany) with an abdominal compression. Motion management was done using PHILIPS bellows (Cleveland, OH) for 4D CT scans. The 4D CT images were acquired with 512×512 pixels at 2 mm slice thickness and 2 mm slice spacing. All phases of DICOM 4D CT datasets were then electronically transferred to the BrainLAB iPlan TPS for contouring purposes. Maximum intensity projection (MIP) and mean intensity projection (average) images were then created in iPlan TPS and auto‐fused with each phase of 4D CT images. Internal target volume (ITV) was delineated on MIP images of the 4D CT scans. PTV was generated from ITV with 5 mm uniform margin with mean volume 36.8±20.7 cc (ranged from 10.0 to 99.9 cc). The critical structures, such as bilateral lungs excluding the ITV (ipsi‐lung), heart, esophagus, and spinal cord, were delineated on the average images of 4D CT scans.

### D. Treatment planning and dose calculation

**Figure 1:**
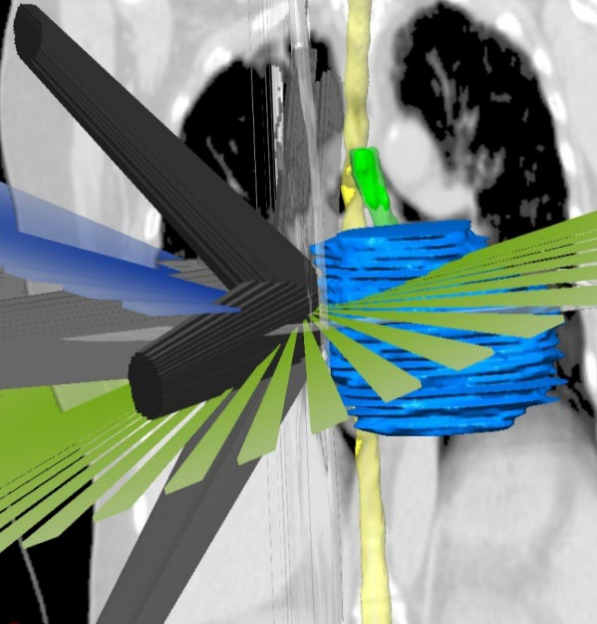
Demonstration of coronal view of noncoplanar conformal arcs and static beams setup with respect to patient anatomy.

Optimal clinical MC SABR treatment plans were generated using a combination of non‐coplanar 3D conformal arcs and beams for the Novalis TX linear accelerator (Varian Palo Alto, CA) with BrainLAB system consisting of high definition MLCs and 6 MV‐SRS (1000MU/min) mode (see Fig. 1). All treatment plans were calculated using XVMC algorithm for heterogeneity corrections with $2.5\times 2.5\times 2.5\;{\text{cm}}^{3}$ dose grid sizes, 2% variance (relative standard deviation of the mean), dose to medium, and accuracy optimized for MLC modeling. All plans had a dose delivery schema of 60 Gy in 5 fractions with at least 95% of the PTV volume received 100% of the prescription dose–DV normalization. For small PTV volume (≤10 cc), a liberal beam margin was used to satisfy the protocol's recommendations to treat at least 3.5 cm beam aperture while keeping the original PTV volume. In cases where the PTV was abutting critical organs, the plans were re‐optimized such that there was no hot spot within the organ that receiving more than 105% of the prescribed dose.

### E. Plan evaluation

The DVHs of all treatment plans were generated in the BrainLAB iPlan TPS and evaluated for the following RTOG 0813 high and intermediate dose spillage dose parameters: ^(4)^

a) R100%: ratio of prescription isodose volume to the PTV (conformality index),
b) R50%: ratio of 50% prescription isodose volume to the PTV,
c) $D_{2\text{cm}}$: maximal dose 2 cm away from PTV in any direction as a percentage of prescription dose, and
d) $V_{20}$: percentage of ipsilateral lung receiving dose equal to or larger than 20 Gy.

Furthermore, all the clinical MC plans were evaluated for the relative volume of normal lung receiving 5 Gy dose, maximum spinal cord dose, dose to <15 cc of heart, and dose to <5 cc of esophagus, as well.

**Figure 2:**
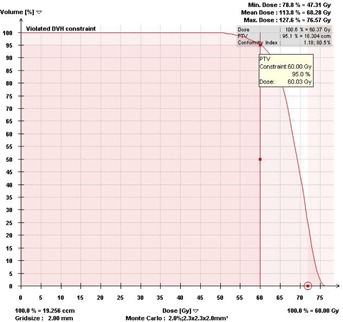
A MC DVH showing 95% of the PTV is conformally covered by the prescription dose (60 Gy). R100%=1.18, mean PTV dose=68.3 Gy, maximum dose=76.6 Gy.

**Figure 3:**
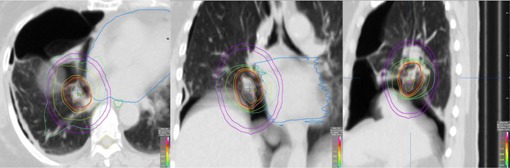
MC Isodose distributions on axial (left), coronal (center) and sagittal (right) views for a SABR lung plan. Lines indicate ITV (innermost), followed by PTV. Higher isodose lines, such as 100%, 95%, and 80%, had sharp falloff, hotspot was within 128%; 20 Gy isodose line restricted mainly in the ipsilateral lung. Pink color ring was contoured to calculate $D_{2\text{cm}}$ (%).

A clinical MC computed DVH for one representative patient is shown in Fig. 2 and MC dose distribution (axial, coronal and sagittal views) for the same patient is shown in Fig. 3.

## III. RESULTS & DISCUSSION

Tables 1–5 present the results of dosimetric parameters from 18 SABR lung plans correspond to RTOG 0813 dosimetry evaluation criteria. Only six out of 18 patients met all the RTOG 0813 compliance criteria. Eight of 18 patients had minor deviations in R100%, four in R50%, and nine in $D_{2\text{cm}}$. However, only one patient (# XIII) associated with the largest PTV volume had minor violation in $V_{20}$. All of the OAR doses, such as maximum cord dose, dose to <15 cc of heart, and dose to <5 cc of esophagus, were satisfactory for RTOG 0813 criteria, except in patient # X, where the dose to <15 cc of heart which had tumor volume abutting heart was higher with minor deviation on RTOG 0813 requirement.

Table 1 shows that eight out of 18 patients had minor deviations in R100%. The mean value of R100% was 1.22±0.07, resulting in a standard deviation of about 6% from the mean value. However, no major deviation was observed. Minor deviations in R100% were clinically accepted by the physicians. The deviations can be explained, primarily, due to underlying characteristic behaviors of the XVMC algorithm for dose distribution prediction, or it may be during initial plan review physician subjective in selecting a treatment plan based on clinical decisions. The underlying characteristic behaviors of XVMC could be explained by more accurate modeling of a) secondary scatter photons, and b) lateral electron equilibrium, specifically at the lung and tumor interfaces.

In Table 2 it will be seen that only four out of 18 patients did not meet the RTOG 0813 R50% criteria. The mean value of R50% was 4.1±0.7. However, one patient who was near the tolerance value (# XIII with the largest PTV volume of nearly 100 cc, which was in the border line range of SABR treatment volume) was deemed clinically acceptable by the physician and treated. Other three patients (# XII, # XIV, and # XVII) who had minor deviations in R50% were clinically accepted by the physicians and were treated. In those three cases, the minor deviations in R50% could be explained by subjectivity of the physician‐dependent initial treatment plan review based on clinical decisions. Also, it could be due to patient geometry and tumor location preventing optimal beam arrangements or some contributions from underlying characteristic behaviors of the XVMC algorithm for dose prediction as discussed earlier.

As reported in Table 3, nine out of 18 patients had minor deviations in RTOG 0813 $D_{2\text{cm}}$ criteria. The mean value of $D_{2\text{cm}}$ was 58.9±8.1, resulting in a standard deviation of about 9% from the mean value. However, no major deviation was observed. Those deviations could be justified by numerous reasons: a) primarily, due to underlying characteristic behaviors of the XVMC algorithm depending in predicting more accurate dose distribution in surrounding low‐density lung and heterogeneous tumor interfaces; b) those minor deviations in $D_{2\text{cm}}$ were mostly associated to large/medium size tumors with a suboptimal conformality index; in general, the larger the tumor volume gets, the harder it is to meet the $D_{2\text{cm}}$ criteria; c) patient‐specific clinical restrictions preventing optimal beam arrangements; d) or during the initial subjective treatment plan review by the physician to respect critical structures dose tolerance.

**Table 1:** Evaluation of R100% in SABR lung plans calculated by iPlan XVMC algorithm

*Patient #*	*PTV Vol. (cc)*	*RTOG 0813 Minor Deviation*	*R100% iPlan MC*
I	19.3	1.2‐1.5	1.18
II	18.9	1.2‐1.5	1.26^a^
III	31.2	1.2‐1.5	1.18
IV	36.2	1.2‐1.5	1.12
V	38.9	1.2‐1.5	1.34^a^
VI	40.0	1.2‐1.5	1.19
VII	40.0	1.2‐1.5	1.18
VIII	44.1	1.2‐1.5	1.25^a^
IX	49.5	1.2‐1.5	1.15
X	62.1	1.2‐1.5	1.30^a^
XI	36.1	1.2‐1.5	1.11
XII	30.3	1.2‐1.5	1.25^a^
XIII	99.9	1.2‐1.5	1.34^a^
XIV	10.0	1.2‐1.5	1.32^a^
XV	22.9	1.2‐1.5	1.15
XVI	46.7	1.2‐1.5	1.19
XVII	20.1	1.2‐1.5	1.25^a^
XVIII	15.8	1.2‐1.5	1.20
AVG	36.8		1.22
STDEV	20.7		0.07

a Data that have minor deviations from RTOG 0813 criteria. PTV=planning target volume; R100%=ratio of prescription isodose volume to PTV; AVG=Average; STDEV=standard deviation.

**Table 2:** Evaluation of R50% in SABR lung plans calculated by iPlan XVMC algorithm

*Patient #*	*PTV Vol. (cc)*	*RTOG 0813 Minor Deviation*	*R50% iPlan MC*
I	19.3	4.56‐5.59	3.7
II	18.9	4.57‐5.61	4.5
III	31.2	4.35‐5.35	3.7
IV	36.2	4.26‐5.26	3.9
V	38.9	4.21‐5.21	4.2
VI	40.0	4.19‐5.19	3.7
VII	40.0	4.19‐5.19	3.7
VIII	44.1	4.11‐5.11	3.6
IX	49.5	4.01‐5.01	2.5
X	62.1	3.70‐4.88	3.2
XI	36.1	4.26‐5.26	3.8
XII	30.3	4.49‐5.37	4.7^a^
XIII	99.9	3.27‐4.34	4.4^a^
XIV	10.0	4.89‐5.89	5.3^a^
XV	22.9	4.48‐5.48	4.4
XVI	46.7	4.05‐5.05	3.6
XVII	20.1	4.54‐5.56	5.5^a^
XVIII	15.8	4.63‐5.69	4.6
AVG	36.8		4.1
STDEV	20.7		0.7

a Data that have minor deviations from RTOG 0813 criteria.

PTV=planning target volume; R50%=ratio of 50% prescription isodose volume to PTV; AVG=Average; STDEV=standard deviation.

**Table 3:** Evaluation of $D_{2\text{cm}}$ in SABR lung plans calculated by iPlan XVMC algorithm

*Patient #*	*PTV Vol. (cc)*	*RTOG 0813 Minor Deviation*	$D_{2\text{cm}}$ *iPlan MC*
I	19.3	52.77‐61.47	52.0
II	18.9	52.59‐61.24	49.0
III	31.2	57.07‐66.83	55.0
IV	36.2	58.55‐69.24	64.0^a^
V	38.9	59.23‐70.76	64.0^a^
VI	40.0	59.50‐71.38	62.0^a^
VII	40.0	59.50‐71.38	53.0
VIII	44.1	60.53‐73.68	55.0
IX	49.5	61.88‐76.72	60.0
X	62.1	64.42‐82.45	66.0^a^
XI	36.1	58.50‐69.13	68.0^a^
XII	30.3	56.67‐66.33	59.0^a^
XIII	99.9	70.48‐89.32	79.0^a^
XIV	10.0	50.00‐58.00	50.0
XV	22.9	54.33‐63.42	59.0^a^
XVI	46.7	61.25‐75.31	61.0
XVII	20.1	53.09‐61.86	61.0^a^
XVIII	15.8	51.36‐59.70	44.0
AVG	36.8		58.9
STDEV	20.7		8.1

a Data that have minor deviations from RTOG 0813 criteria.

PTV=planning target volume; $D_{2\text{cm}}=\text{maximal dose}$ 2 cm from PTV in any direction as a percentage of prescription dose; AVG=Average; STDEV=standard deviation.

Tables 4 and 5 report the dosimetric evaluation of normal lung, $V_{20}$ (Table 4) and $V_{5}$, as well as other OARs doses (Table 5) such as dose to heart, maximum cord dose, and esophagus doses in SABR lung plans calculated by iPlan XVMC algorithm respectively.

The results of this study showed that the RTOG 0813 high‐dose spillage criteria, such as R100%, was met only in 10 out of 18 patients, with 8 minor deviations. The standard deviation of R100% from the mean value was about 6%. However, there was no major deviation in R100%. One of the intermediate dose spillage criteria, R50% passed the RTOG guidelines for 14 patients, with only three minor deviations and one major deviation, near the tolerance value. It was observed that the major deviation was due to the largest PTV volume that was outside or near the recommended tolerance of SABR treatment volume. Other three patients (patient # XII, # XIV, and # XVII) who had minor deviations in R50% had suboptimal beam arrangements due to patient geometry and tumor locations. All those plans were clinically accepted by the physicians and were treated. However, 50% of our patients had minor deviations in $D_{2\text{cm}}$, but no major deviation was observed. The mean value of $D_{2\text{cm}}$ was 58.9±8.1, with a standard deviation resulting from the mean value of about 9%. Only one patient, who had the largest PTV volume, had minor deviation from RTOG 0813 criteria in normal lung $V_{20}$. No minor deviations from RTOG 0813 criteria were observed for all other OAR dose tolerances such as maximum cord dose, dose to <15 cc of heart, and dose to <5 cc of esophagus, except for one patient (patient #X) whose tumor was next to the heart.

**Table 4:** Evaluation of normal lung $V_{20}$ in SABR lung plans calculated by iPlan XVMC algorithm

*Patient #*	*PTV Vol. (cc)*	*RTOG 0813 Minor Deviation*	$V_{20}(\%)$ ^a^ *iPlan MC*
I	19.3	10‐15	3.6
II	18.9	10‐15	5.7
III	31.2	10‐15	4.3
IV	36.2	10‐15	3.8
V	38.9	10‐15	6.1
VI	40.0	10‐15	6.8
VII	40.0	10‐15	8.5
VIII	44.1	10‐15	3.5
IX	49.5	10‐15	7.2
X	62.1	10‐15	6.6
XI	36.1	10‐15	2.1
XII	30.3	10‐15	7.3
XIII	99.9	10‐15	13.8^b^
XIV	10.0	10‐15	1.1
XV	22.9	10‐15	3.7
XVI	46.7	10‐15	5.7
XVII	20.1	10‐15	2.5
XVIII	15.8	10‐15	2.6
AVG	36.8		5.3
STDEV	20.7		3.0

a Normal lung $V_{20}$ values ranged from 1.1% to 13.8% (mean=5.3±3.0%).

b No minor deviation in $V_{20}$ from the RTOG 0813 criteria was observed; except for one patient (patient # XIII) whose PTV volume was about 100 cc and whose $V_{20}=13.8\%$. PTV=planning target volume; Ipsi‐lung=Ipsilateral lung; $V_{20}=\text{percentage of ipsilateral lung}$ receiving dose equal to or larger than 20 Gy; AVG=Average; STDEV=standard deviation.

Minor deviations from RTOG compliance criteria were also reported in previous studies.^5^, ^6^ For instance, Rana et al.^(6)^ compared the treatment plans computed by analytic anisotropic algorithm (AAA) and Acuros XB for the same number of MUs. It was reported that AAA plans had higher R100%, R50%, and $D_{2\text{cm}}$ when compared to the Acuros XB plans. However, $V_{20}$ of lung was found to be lower in AAA plans. Rana and colleagues also reported minor deviations in R100%, R50%, and $D_{2\text{cm}}$ for both the AAA and Acuros XB plans, with Acuros XB showing minor deviation in fewer cases when compared to AAA. In that study, it was also reported lower PTV coverage using Acuros XB when compared to AAA. On the other dosimetric study reported by Li et al.^(5)^ using Monaco MC algorithm in XiO TPS also observed minor deviations from RTOG 0813 compliance criteria on R50%, R100%, and $D_{2\text{cm}}$. Our findings were consistent with those previous reports.

One of the differences between our study and Rana et al.^(6)^ is the plan normalization method. Dosimetric results from their study were based on the normalization at a single point per RTOG requirement, whereas our results are based on DV normalization technique. In this preliminary study, we have demonstrated the feasibility of using DV normalization technique to replicate/satisfy RTOG 0813 criteria. More clinically relevant DV normalization techniques have allowed us to investigate the dosimetric impact of the tumor size‐dependent radiobiological effectiveness of the delivered MC dose to 99% of PTV volume versus local control.^(19)^ We plan to continue building a MC lung SABR patient's database in our clinic and further investigate RTOG 0813 dosimetric parameters, as well as it's radiobiological effectiveness. In the future, further studies involving a large cohort of patients, including central as well as peripheral lung tumors, are needed. This will help to establish new parameters specific for the MC‐based dose calculations in the lung SABR planning. Also, the dosimetric impact of various plan normalization techniques on RTOG 0813 compliance criteria need to be further investigated.

**Table 5:** Evaluation of normal lung $V_{5}$, dose to heart, maximum cord dose, and esophagus doses in SABR lung plans calculated by iPlan XVMC algorithm

*Patient #*	*PTV Vol. (cc)*	*Normal Lung* $V_{5}$ ^a^ *(%)*	*Maximum Dose to Spinal Cord* ^b^ *(Gy)*	*Dose to* <15cc *of Heart* ^c^ *(Gy)*	*Dose to* <5cc *of Esophagus* ^d^ *(Gy)*
I	19.3	21.8	13.6	23.4	2.9
II	18.9	22.2	21.4	16.2	15.8
III	31.2	14.5	5.7	22.4	3.6
IV	36.2	11.5	5.5	4.5	4.3
V	38.9	19.1	21.5	5.6	16.4
VI	40.0	16.3	5.1	31.5	3.5
VII	40.0	28.0	11.4	20.5	10.4
VIII	44.1	10.3	5.5	31.4	5.8
IX	49.5	22.5	11.4	15.7	6.5
X	62.1	22.1	5.4	42.6^e^	5.2
XI	36.1	3.9	9.2	4.1	14.0
XII	30.3	8.4	6.5	6.2	4.8
XIII	99.9	31.5	18.5	5.9	16.1
XIV	10.0	7.6	18.4	10.9	2.5
XV	22.9	11.3	19.3	11.8	13.2
XVI	46.7	15.4	24.5	7.8	11.1
XVII	20.1	4.7	11.4	2.1	5.8
XVIII	15.8	11.7	9.1	1.2	3.9
AVG	36.8	15.7	12.4	14.7	8.1
STDEV	20.7	7.8	6.6	11.8	5.0

a Normal lung $V_{5}$ values ranged from 3.9% to 28% (mean=15.7±7.8%).

b RTOG minor deviation criteria for maximum cord dose >30 Gy.

c RTOG minor deviation criteria for dose to <15cc of heart >32 Gy,

d RTOG minor deviation criteria for dose to <5cc of esophagus >27.5 Gy.

e No minor deviation from RTOG 0813 criteria was observed; except for one patient (patient # X) whose tumor was abutting heart. PTV=planning target volume; $V_{5}=\text{percentage of ipsilateral lung}$ receiving dose equal to or larger than 5 Gy; AVG=Average; STDEV=standard deviation.

## IV. CONCLUSIONS

The preliminary dosimetric results for our limited iPlan XVMC dose calculations algorithm indicates that not all patient's plans could meet the dosimetric guidelines set by RTOG 0813 protocol. Minor deviations in R100%, R50%, and $D_{2\text{cm}}$ were observed in the majority of the patients (i.e., 2/3) in one way or another. When using an exclusive highly sophisticated iPlan XVMC algorithm for dose calculations, the RTOG 0813 dosimetric compliance criteria, such as R100% and $D_{2\text{cm}}$, may need to be reexamined. Based on our limited number of patient datasets, in general, R100% and $D_{2\text{cm}}$ criteria could be relaxed by about 6% and 9%, respectively, to pass the RTOG 0813 dosimetric criteria in most of those patients. More patient plans need to be studied to make recommendation for R50% criteria. No adjustment is required for normal lung $V_{20}$, and other OAR dose tolerances such as maximum cord dose, dose to <15 cc of heart, and dose to <5 cc of esophagus when exclusively using MC‐based dose calculations. In order to establish new MC‐specific dose parameters, further investigation with a large number of patients, including peripheral lung tumors, is anticipated and highly recommended.

## ACKNOWLEDGMENTS

The first author of this paper would like to express his sincere gratitude to Suresh Rana, MS, of ProCure Proton Therapy Center, Oklahoma City, for many stimulus discussions while working on this research project and also for his editorial help in preparing this manuscript.
